# Matters to Be Discussed

**Published:** 1916-06-10

**Authors:** 


### THE COLLEGE OF NURSING MEETING.

### MATTERS TO BE DISCUSSED.

THE following communication has been sent by the Hon. Arthur Stanley, M.P., M.V.O., Chairman of the College of Nursing, Limited, to the representatives nominated to the Consultative Board:—

THE COLLEGE OF NURSING, LIMITED.
83 Pall Mall, London, S.W.,
June 7, 1916.

DEAR SIR (OR MADAM),—The Council has decided to invite all those who have been nominated by the Nurse-Training schools of Hospitals or Poor-Law infirmaries throughout the country as representatives upon the Consultative Board of the College to a meeting to be held on Thursday, June 15, at 3 P.M., in the Great Hall of St. Thomas's Hospital, kindly lent by the Governors for the occasion.

Amongst the topics which the Council proposes for discussion are the following:—

1. The first draft of a Bill for the Registration of Nurses to be promoted by the College.

2. The formation of the first Register of Members of the College, and the general conditions for the admission to it of nurses now in practice. (Leaflet enclosed.)

3. The constitution of the Consultative Board, whether—

(*a*) As a large body, say 450 or 500, meeting, at the present stage of the College, frequently for the consideration of the general questions relating to curriculum and the recognition of nurse-training schools, and, later, meeting usually once a year at a large town either in England, Wales, Scotland, or Ireland, for the reading and discussion of papers bearing on the training of nurses, and cognate educational subjects; or—

(*b*) As a smaller body, say 100, meeting more frequently than would be practicable with the larger number with a view to the examination in greater detail of the proposals put forward by the Consultative Committee.

I shall be obliged if you will state on the postcard sent herewith whether you propose to be present, so that arrangements may be made for tea after the meeting.—Yours faithfully,

ARTHUR STANLEY, Chairman.

P.S.—In order to avoid confusion, and to give the fullest opportunities for full discussion by the meeting, I propose to ask speakers to address the audience from the platform, after I have announced their names, and to limit their speeches as nearly as possible to five minutes.

